# Lichen Polyphenolic Compounds for the Eradication of *Candida albicans* Biofilms

**DOI:** 10.3389/fcimb.2021.698883

**Published:** 2021-09-16

**Authors:** Marion Girardot, Marion Millot, Guillaume Hamion, Jeanne-Louise Billard, Camille Juin, G M A Ndong Ntoutoume, Vincent Sol, Lengo Mambu, Christine Imbert

**Affiliations:** ^1^UMR CNRS 7267, Laboratoire Ecologie et Biologie des Interactions, Université de Poitiers, Poitiers, France; ^2^EA 7500, Laboratoire PEIRENE, Université de Limoges, Limoges, France

**Keywords:** depsides, lichens, biofilm, *Candida albicans*, evernic acid

## Abstract

Lichens, due to their symbiotic nature (association between fungi and algae), constitute a chemical factory of original compounds. Polyphenolic compounds (depsides and depsidones) are the main constituents of lichens and are exclusively biosynthesized by these organisms. A panel of 11 polyphenols was evaluated for their anti-biofilm activity against *Candida albicans* biofilms on the maturation phase (anti-maturation) (MMIC_50_) as well as on preformed 24-h-old biofilm (anti-biofilm) (MBIC_50_) using the XTT assay. Minimum inhibitory concentrations of compounds (MICs) against *C. albicans* planktonic yeast were also determined using a broth microdilution method. While none of the tested compounds were active against planktonic cells (IC_50_ > 100 µg/ml), three depsides slowed the biofilm maturation (MMIC_50_ ≤12.5 µg/ml after 48 h of contact with *Candida* cells). Evernic acid was able to both slow the maturation and reduce the already formed biofilms with MBIC_50_ ≤12.5 µg/ml after 48 h of contact with the biofilm. This compound shows a weak toxicity against HeLa cells (22%) at the minimal active concentration and no hemolytic activity at 100 µg/ml. Microscopic observations of evernic acid and optimization of its solubility were performed to further study this compound. This work confirmed the anti-biofilm potential of depsides, especially evernic acid, and allows to establish the structure–activity relationships to better explain the anti-biofilm potential of these compounds.

## Introduction

*Candida albicans* is a common microorganism which colonizes the gastrointestinal and reproductive tracts and oral cavity of humans (Underhill and Iliev, 2014; Nobile and Johnson, 2015). Sometimes, especially with elderly and/or frail patients (immunocompromised, with long-term stays in intensive care units, after digestive surgery, *etc*.), *C. albicans* becomes an opportunistic agent leading to superficial or invasive infections (Pappas, 2006; Wisplinghoff et al., 2014; Campion et al., 2015). In these cases, *Candida* biofilms may occur on catheter, dental, and/or mucosal surfaces. The first step for the colonization and biofilm formation is the adherence of *Candida* yeasts onto the host surfaces (Radford et al., 1999; Soustre, 2004; Bürgers et al., 2010). Adhesion is followed by biofilm maturation, which is characterized by the production of hyphae and an extracellular matrix (Chandra et al., 2001). Biofilm formation is implicated in 80% of infections and is an important virulence factor. Indeed sessile cells become less susceptible to both antimicrobial medicines and host immune responses (Donlan and Costerton, 2002). Biofilms are specially resistant to convential antifungal drugs, and the therapeutic options are limited. Therefore, the development of new drugs is crucial to enrich the list of anti-*Candida* drugs active against biofilms, which is currently limited to the lipid formulation of amphotericin B and echinocandins.

Lichen metabolism results from the performing cooperation between an alga and/or cyanobacterium and a fungus. Using photosynthesis, the algae and/or cyanobacterium transfers simple sugars to the fungus which thus becomes able to biosynthesize complex and specific metabolites. These metabolites are responsible for lichen defenses against environmental stress and are endowed with several biological activities (Boustie and Grube, 2005; Brandão et al., 2013; Sweidan et al., 2017). With lichens living in harsh conditions (exposed to strong UV radiation and low temperatures) and being colonized by a wide range of microbial species, the metabolites they produce are good weapons—for example, *Rhizocarpon geographicum*, a lichen which grow in sun-exposed rocks, biosynthesize rhizocarpic acid in order to filter UV radiation (Varol, 2018). The wolf lichen, *Letharia vulpina*, produces high quantities of vulpinic acid, which is toxic for its predators (Lauterwein et al., 1995). Nevertheless, lichens are slow-growing organisms, and numerous species are scarce, leading to difficult access to secondary metabolites. While lichen extracts are increasingly studied for their antimicrobial properties (Mitrovic et al., 2014), their specific metabolites are still poorly investigated.

To our knowledge, only three lichen compounds have been the object of anti-biofilm activity studies as reported in the literature. The common dibenzofuran-like metabolite, (+)‐usnic acid, has been tested against several bacterial or fungal biofilms: *Streptoccocus pyogenes*, *Staphylococcus aureus*, *Pseudomonas aeruginosa*, and *Candida* sp. biofilms (Francolini et al., 2004; Kvasnickova et al., 2015; Nithyanand et al., 2015a). Chang *et al*. demonstrated the activity of retigeric acid B, a pentacyclic triterpenoid isolated from the lichen *Lobaria kurokawae* (Chang et al., 2012). This compound inhibited *C. albicans* yeast-to-hypha transition in infected mice and acted synergistically with azoles to block *C. albicans* biofilm formation (Chang et al., 2012). Nomura et al. reported a moderate effect of depsides and related volatile compounds against *Legionella pneumophila* biofilms (Nomura et al., 2013). Besides this, the modulating effect of evernic acid on quorum sensing of *P. aeruginosa* has also been described, encouraging thorough research investigations on depsides (Gökalsın and Sesal, 2016).

Thus, lichens appear to be an unexplored non-negligible source of anti-biofilm compounds targeting both bacteria and fungi. In a preliminary study, 38 lichen species were screened for their antimaturation and antibiofilm activity (Millot et al., 2017). The biological tests revealed that most of the lichen extracts endowed with an antibiofilm activity contain large amounts of depsides. To deeply explore the potencies of such metabolites as new anti-biofilm candidates, 11 lichen compounds were tested against *C. albicans* in planktonic and biofilm conditions. Cytotoxic activities have also been evaluated (hemolysis and growth inhibition on HeLa cells). Further evaluations were performed on the most active compound, evernic acid.

## Materials and Methods

### Lichen Compounds

Evernic acid (**1**) was obtained from *Evernia prunastri* and (+)-usnic acid (**11**) and thamnolic acid (**3**) from *Usnea florida* (Dieu et al., 2020). Squamatic acid (**2**) came from *Cladonia squamosa*, atranorin (**4**) from *Pseudevernia furfuracea*, gyrophoric acid (**5**) from *Lasallia pustullata*, norstictic acid (**6**) from *Pleurosticta acetabulum* (Delebassée et al., 2017), salazinic acid (**7**) from *Parmelia saxatilis*, stictic acid (**8**) from *Xanthoparmelia conspersa*, and physodic and 3-hydroxyphysodic acids (**9**–**10**) from *Hypogymnia tubulosa*. The lichens were firstly macerated with acetone at 5% (m/v). After filtration and concentration of the solvent under vacuum, the liquid extracts were centrifugated (5 min at 3,000 rpm). After centrifugation, the resulting precipitate was rinsed two times with fresh acetone and submitted again to centrifugation to give pure compounds **1**–**2** and **4**–**8** (Delebassée et al., 2017; Dieu et al., 2020). For the lichen *U. florida*, the precipitate obtained after centrifugation was rinsed with CH_2_Cl_2_ and gave a supernatant (compound **11**) and a second precipitate (compound **3**). Compounds **9**–**10** were obtained from the dried acetonic extract of *Hypogymnia physodes* (100 mg) submitted to centrifugal radial chromatography eluted by a mixture of toluene and ethyl acetate (gradient solvent from 90:10 to 50:50). The purity of lichen compounds was checked by HPLC analyses on Waters Alliance 2690 using a reversed-phase Hibar^®^ LiChrospher^®^ 100 C-18 column (5 µm, 250 × 4 mm, Merck) coupled to a photodiode-array detector (Waters 996). The elution solvent MeOH–H_2_O–H_3_PO_4_ at 80:20:0.9 was used in an isocratic mode at a flow rate of 1 ml/min according to the protocol of Yoshimura et al. (1994).

### Biological Activities

#### Minimal Inhibitory Concentration

Stock solutions of lichen compounds were prepared in DMSO at 10 mg/ml and were used for all the experiments performed. Their antifungal minimum inhibitory concentrations (MICs) were determined by a broth microdilution method (CLSI reference M27-A3 micromethod adapted protocol) as previously described (Millot et al., 2017). Briefly, an inoculum of *C. albicans* ATCC^®^ 28367™ (American Type Culture Collection) adjusted to 0.5 Mac Farland absorbance was diluted 1:1,000 in RPMI-MOPS. A volume of this yeast culture was added to an equivalent volume of compound solution at several concentrations (100 to 0.048 μg/ml final) in 96-well microtiter plates. Controls as untreated yeasts (negative control), yeasts treated with 2% DMSO and yeasts treated with fluconazole (positive control) were included. After 24 and 48 h incubation at 37°C without shaking, the MICs were determined. All tests were performed in duplicate in at least two separate experiments. Lichen compounds with a MIC less than 50 μg/ml were considered as potentially useful.

#### Anti-Maturation and Anti-Biofilm Tests

The anti-maturation and/or anti-biofilm activities of lichen compounds were assessed as previously described (Millot et al., 2017). Briefly, *C. albicans* (ATCC^®^ 28367™) cultures were prepared at a final concentration of 1 × 10^7^ cells/ml in yeast nitrogen base medium (Difco, Detroit, MI), supplemented with 50 mM glucose (Sigma, St Louis, MO) (YNB-Glc) from a preculture incubated overnight at 37°C without shaking.

A total of 200 µl of yeast culture was added to each well of untreated 96-well tissue culture plates. The plates were then incubated at 37°C for 2 h for the anti-maturation test and for 24 h for the anti-biofilm test. After aspiration of spent media and free-floating microorganisms, 250 μl per well of YNB-Glucose and 50 μl lichen compound samples obtained by serial twofold dilutions of each stock solution over the final range 200 to 0.097 μg/ml or 200 to 6.25 μg/ml were added to wells. Controls were also present: untreated yeasts (negative control) and yeasts treated with 2% DMSO. After 24 and/or 48 h of incubation at 37°C followed by aspiration of spent media and free-floating microorganisms, the wells were washed with phosphate-buffered saline (PBS) and observed under an inverted optical microscope (IX51^®^ inverted microscope, Olympus). Biofilms were quantified using a metabolic assay based on the reduction of a tetrazolium salt (XTT) and absorbance measurements at 492 nm (microplate reader LP400; Sanofi Diagnostics Pasteur) (Cocuaud, 2005; Dalleau et al., 2008). The percent inhibition was determined from the absorbance values compared to the mean absorbance value obtained for untreated wells (100%).

Concentrations of lichen products inhibiting 50% of biofilms were determined for each tested sample by constructing a dose–response curve and selecting the closest tested concentration value above or equal to 50% inhibition. All anti-maturation and anti-biofilm tests were performed at least in triplicate in at least two separate experiments. The lichen compounds with MMIC_50_ or/and MBIC_50_ less than 100 μg/ml were considered as potentially useful.

#### Statistical Analysis

The differences among the test groups were determined by Kruskal–Wallis tests (biostaTGV website: https://marne.u707.jussieu.fr/biostatgv/?module=tests).

#### Hemolysis Test

First, several concentrations of each stock solution were prepared by serial twofold dilutions in physiological serum over the final range 100 to 1.56 μg/ml (the final DMSO concentrations did not exceed 1% of the overall volume in wells). Then, 480 µl of each dilution was added to 20 µl of healthy red blood cells (Poitiers University Hospital, Hematology Department) in microtubes (final hematocrit = 4%). Some microtubes were reserved for controls: untreated red blood cells (negative control), cells treated with sterile distilled water (positive control = 100% hemolysis), and cells treated with 1% DMSO. After 1 h of incubation at 37°C, the tubes were centrifuged for 10 s at 14,000 *g* (A14 centrifuge Jouan) twice, and 200 µl of each supernatant was transferred into 96-well microtiter plates for colorimetric measurements at 450 nm (microplate reader LP400; Sanofi Diagnostics Pasteur) in order to determine the hemolysis percentages of each compound (Evans et al., 2013). All tests were performed in duplicate (at least two separate experiments).

#### Growth Inhibition of HeLa Cells

HeLa human epithelial cervix cells were grown as monolayers at 37°C in a 5% CO_2_–95% air humidified atmosphere in RPMI supplemented with 10% FBS and 10 mg/ml gentamycin and streptomycin. Cells (2,000 cells/well) were seeded into each well of 96-well plates. After 24 h of incubation, 50 µl of lichen molecules, solubilized in DMSO before being diluted to 100 μg/ml final concentration in cell culture medium in microplates or to the lowest anti-biofilm or anti-maturation IC_50_ obtained (12.5 or 6.25 μg/ml) for the three most active compounds, was added in each well. The final DMSO concentration was less than 1% and tested as a negative control. Cell viability at 72 h was studied using the MTT assay. Then, 5 µl of MTT (10 mg/ml) was added to each well, and the plates were incubated for 4 h. The medium was then removed by aspiration, and 200 µl DMSO was added to each well. Proliferation was determined spectrophotometrically by measuring the absorbance at 540 nm. The percent of growth inhibition (%GI) was calculated as follows: %GI = [(DO (ʎ_540 nm_) control - DO (ʎ_540 nm_) treated)/DO (ʎ_540 nm_) control] * 100. All experiments were performed in duplicate with at least two replicate experiments.

#### Anti-Biofilm Effect of Evernic Acid by SEM

The anti-biofilm protocol was similar to that previously described except for the fact that the biofilms were formed on polycarbonate (RD128-PC) coupons placed on well bottoms of Corning ^®^Costar ^®^24-well polystyrene microplates (Corning Inc., USA). Furthermore, 1.2 ml *C. albicans* (ATCC ^®^28367™) suspension at 1 × 10^7^ cells/ml was added to the wells. The microplates were then incubated at 37°C for 24 h. After aspiration of spent media and free-floating microorganisms, evernic acid solubilized in DMSO was added at 50- and 100-μg/ml final concentrations (DMSO did not exceed the 1% final volume) and completed to 1.2 ml per well with YNB-Glucose. Controls were also present: untreated yeasts (negative control) and YNB-Glucose without yeast. After 48 h of incubation at 37°C and then aspiration of spent media and free-floating microorganisms, the wells containing coupons were washed with PBS and cooled in a nitrogen cloud in LEICA EM VCM. This step of cryofixation lasted 30 s, with nitrogen vapors ranging between -150 and -140°C. Leica EM VCT 500 (temperature of liquid nitrogen and under primary vacuum) was used to transfer the samples to the LEICA EM ACE600 system in which a sublimation step was carried out: the temperature was returned to -80°C for 7 min in order to remove any ice crystals, and a 3-nm platinum film was sprayed on the sample in order to make the surface conductive to electrons. With the VCT 500, under vacuum, and at a temperature of at most -140°C, the metallized sample was transferred to the cryo-stage of the THERMOFISHER Volumescope microscope SEM for observation at -100°C.

#### Evernic Acid–Cyclodextrin/Cellulose Nanocrystal Synthesis

The association of cyclodextrine (CD)/cellulose nanocrystal complex (CNC) was obtained and characterized according to the procedure described by our research group (Ndong Ntoutoume et al., 2016). Evernic acid (600 µg), previously dissolved in 100 μl DMF, was introduced into 6 ml of CD/CNC aqueous solution. After 5 min of sonication, the mixture was left without stirring for 24 h. The mixture was centrifuged (13,000 rpm for 10 min), and the pellets were redispersed in 6 ml of saline. The inclusion rate was evaluated by UV–visible spectroscopy (*λ* = 305 nm) after drying and DMF extraction of drug. The resulting evernic acid–CD/CNC complexes were obtained with a 220-μM drug concentration (8.0% by weight relative to CNCs).

Anti-maturation and anti-biofilm tests were performed with *C. albicans* ATCC^®^ 28367™, as previously described, in 96-well microplates with evernic acid alone, cyclodextrin alone, and evernic acid included in cyclodextrin at over 50 to 1.5 µM final range of evernic acid (serial twofold dilution).

## Results

### Lichen Compound Description

**Figure 1:**
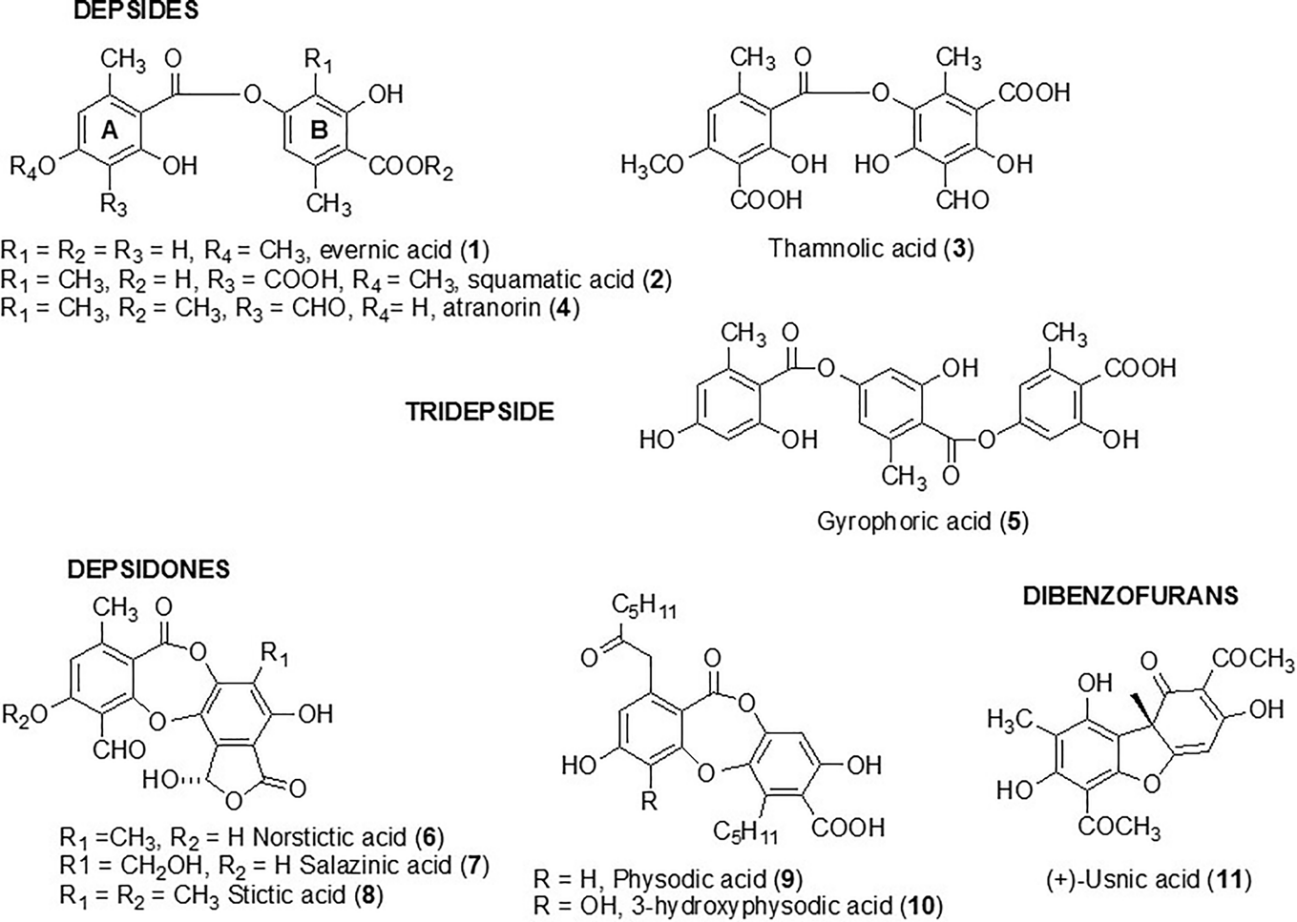
Structure of metabolites tested against biofilm *Candida albicans* yeast.

Four depsides (evernic, squamatic, thamnolic acids, and atranorin) (compounds **1** to **4**) were investigated in the present study. Compounds **1**–**3** were chosen following a preliminary study which screened the activity of 38 lichen species and revealed large amounts of these depsides in the lichen extracts displaying the most promising anti-biofilm activities (Millot et al., 2017). As depsides seemed to be relevant anti-biofilm candidates, we investigated another depside, atranorin (**4**), commonly present in several lichen species (like *Pseudevernia furfuracea*) (Studzinska-Sroka et al., 2017), as well as a tridepside, gyrophoric acid (**5**). Five depsidones, which were also suspected to be responsible for the anti-maturation activity observed in our previous study, were included in the screening. Thus, norstictic, salazinic, and stictic acids (**6**–**8**) as well as physodic and hydroxyphysodic acids (**9**–**10**) were evaluated (**Figure 1**). Finally, usnic acid (**11**), which is the most commonly studied lichen compound and for which some anti-biofilm data are already available, was included to this screening (Kvasnickova et al., 2015; Nithyanand et al., 2015a).

These compounds were evaluated for their anti-maturation and anti-biofilm activities against *C. albicans*. Antifungal activity as well as cytotoxicity towards erythrocytes and HeLa cells was investigated for the most active compounds.

### Activity Against Biofilm Maturation

The aim was to determine the capacity of selected lichen compounds to limit biofilm growth and maturation by adding them just after the yeast adherence step. The minimal maturation inhibition concentration (MMIC) and the minimal biofilm inhibitory concentration (MBIC) obtained with the 11 polyphenolic lichen compounds are presented in **Table 1**. Only three compounds (**1**–**3**), all depsides, displayed anti-maturation activities, with MMIC_50_ less than 35 µM (18, 32, and 30 µM respectively) after 48 h of incubation. The depside atranorin as well as all tested depsidones displayed MMIC_50_ values (≥100 µg/ml) that were too high to be considered as active against the maturation phase.

**Table 1:** Anti-maturation and anti-biofilm activities of 11 lichen compounds against *Candida albicans* expressed as 50% inhibitory concentration (*p* < 0.05) at 24 and 48 h of treatment. IC_50_ is expressed in micromolar for values <100 µg/ml.

Compounds	Anti-maturation activity		Anti-biofilm activity	
	MMIC_50_		MBIC_50_	
	µg/ml (µM)		µg/ml (µM)	
	24 h	48 h	24 h	48 h
**1**	100	6.25 (18)	200	12.5 (37.5)
**2**	50 (128)	12.5 (32)	>200	>200
**3**	50 (120)	12.5 (30)	>200	>200
**4**	>200	>200	>200	>200
**5**	>200	100	>200	>200
**6**	>200	>200	>200	>200
**7**	nd	nd	>200	>200
**8**	200	200	>200	>200
**9**	>200	>200	>200	>200
**10**	>200	100	>200	100
**11**	>200	>200	>200	>200

These experiments were performed in triplicate with at least two replicate experiments.

### Anti-Biofilm Activity

The anti-biofilm activity of lichen compounds was evaluated on a 24-h-old biofilm. Similar to the anti-maturation results, no anti-biofilm activity was observed for dibenzofuran-like usnic acid and depsidones (MBIC_50_ ≥ 100 µg/ml) (**Table 1**).

An anti-biofilm effect was clearly observed for depside **1** (evernic acid) with an activity effective after 48 h of contact (MBIC_50_ < 40 µM).

### Antifungal Activity

The activity of the depsides (**1**–**5**) as well as usnic acid (**11**) was tested against *C. albicans* planktonic yeast. None of the compounds showed antifungal activity with MICs >100 µg/ml (fluconazole MIC: 4 µg/ml).

### Cytotoxic Activity

#### Hemolytic Activity

The tested compounds used at 100 µg/ml did not induce (0%) or induced very weak (between 0.4 and 4%) hemolysis of red blood cells (**Table 2**). Their IC_50_ hemolysis was thus greater than 100 µg/ml and is not given.

**Table 2:** Cytotoxic activity of five depsides (**1**-**5**) expressed as hemolytic percentages at the highest tested concentration (100 µg/ml) and growth inhibition of HeLa cells.

Compounds	Hemolytic activity (%)	Growth inhibition of HeLa cells (% inhibition)		
	at 100 µg/ml	at 6.125 µg/ml	at 12.5 µg/ml	at 100 µg/ml
**1**	0	22.2 ± 2.8	nd	88.0 ± 2.1
**2**	0.4 ± 0.01	nd	14.8 ± 1.6	44.5 ± 6.5
**3**	4.0 ± 0.02	nd	11.0 ± 0.9	45.3 ± 3.5
**4**	0	nd	nd	84.5 ± 6.8
**5**	0.5 ± 0.27	nd	nd	70.0 ± 2.3

nd, not determined.

The results are expressed as mean of inhibition percentages ± standard deviations (SD) depending on the tested concentrations of the compounds (100, 12.5, or 6.25 µg/ml). All experiments were performed in duplicate with at least two replicate experiments.

#### Growth Inhibition of HeLa Cells

Growth inhibition was evaluated for depsides that were the most active against *Candida* biofilm. Two concentrations were tested: 100 µg/ml and the lowest anti-biofilm or anti-maturation IC_50_ (12.5 or 6.25 μg/ml) for depsides **1**–**3**. The results are presented in **Table 2**.

At the lowest tested concentrations, corresponding to those with anti-biofilm activity, the maximum inhibition of HeLa cell growth was 22% for compounds **1**–**3**. At a concentration of 100 µg/ml, squamatic and thamnolic acids (**2** and **3**) induced the lowest growth inhibition (about 44 *versus >*70% for the other selected compounds), while evernic acid (1) is highly cytotoxic. These results are in accordance with a previous study (Kizil et al., 2014). Furthermore, according to Shcherbakova et al. (2021), a prediction of cytotoxicity risks of evernic acid reports the absence of mutagenic, tumorigenic, reproductive, and irritant effects.

### Additional Studies With Evernic Acid

#### Scanning Electronic Microscopy

**Figure 2:**
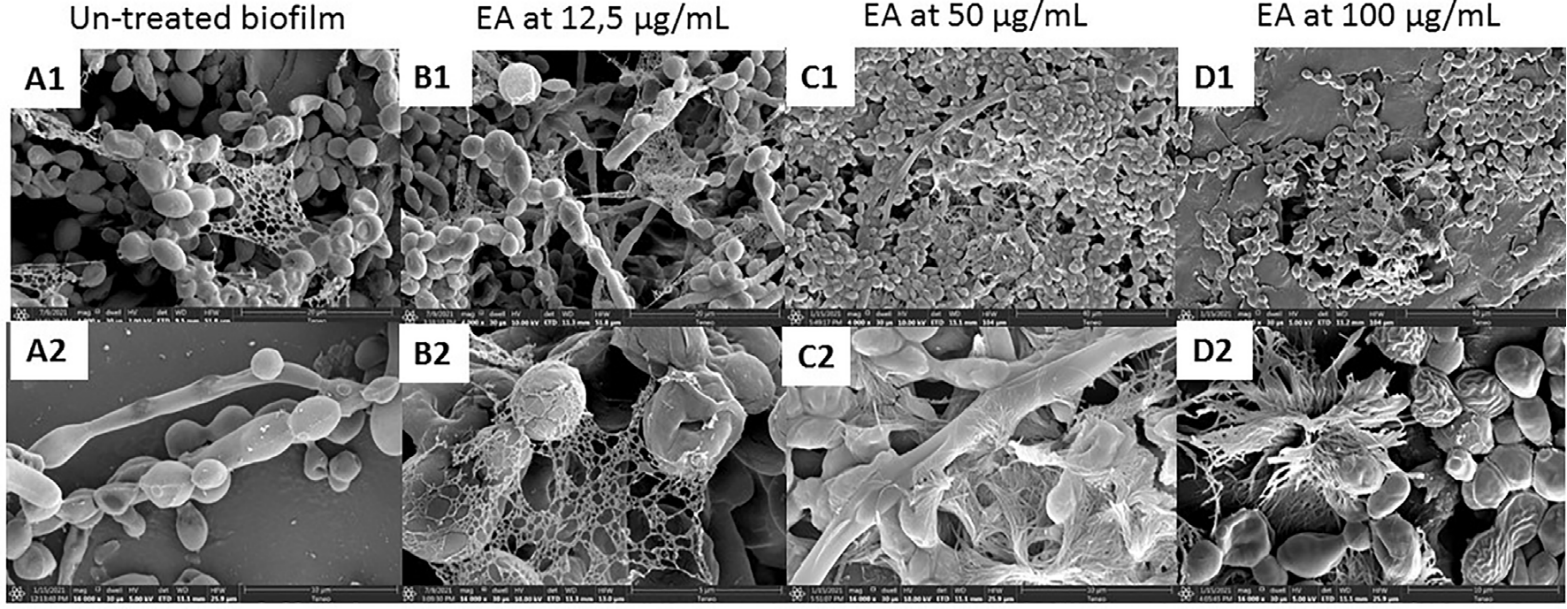
*Candida albicans* biofilm observed by scanning electron microscopy. *C. albicans* biofilm without evernic acid (**A**, 1, 2). *C. albicans* biofilm treated with 12.5 µg/ml (**B**, 1, 2), 50 µg/ml (**C**, 1, 2), and 100 µg/ml (**D**, 1, 2) evernic acid. (**A**—1, **B**—1, **C**—1, **D**—1: magnification, ×4,000; **A**—2, **B**—2, **C**—2, **D**—2: magnification, ×16,000).

The effects of the most active compound, evernic acid, on a 24-h *C. albicans* ATCC ^®^28367™ biofilm were observed by SEM after 48 h of incubation at three concentrations (12.5, 50, and 100 µg/ml) (**Figure 2**).

SEM observations confirmed the presence of yeast forms, hyphae, and matrix forming the *C. albicans* biofilms (**Figures 2, A1, 2**). Treated biofilms with concentrations up to 50 µg/ml showed an altered yeast cell morphology as well as a modification of the extracellular matrix distribution. After the treatment, some yeasts were wrinkled, and the matrix was more condensed with a less coating aspect. These modifications were observed with 50 µg/ml (150 µM) evernic acid (**Figures 2, B1, 2**) and were more visible with 100 µg/ml (300 µM) evernic acid (**Figures 2, C1, 2**). However, no significant effects were observed when the biofilm is treated with 12.5 µg/ml of evernic acid, and the effect of the lichen compound on matrix could not be assessed.

#### Anti-Maturation and Anti-Biofilm Effects of Evernic Acid Included in Cyclodextrin

**Figure 3:**
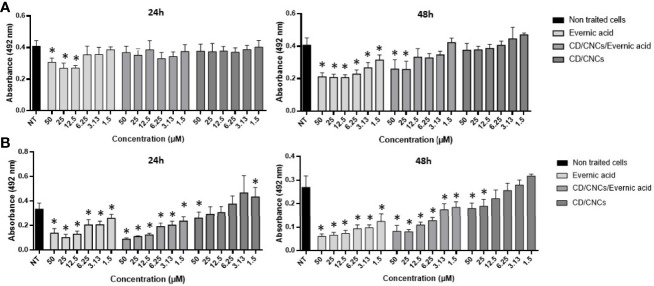
Anti-maturation **(A)** and anti-biofilm **(B)** effects of evernic acid included or not in cyclodextrin (CD/CNCs) after 24 and 48 h of treatment against *Candida albicans* ATCC^®^ 28367™. The results are expressed as mean of absorbances ± SD depending on the tested concentrations of the samples. NT, non-treated cells (YNB-Glc control). The asterisk denotes inhibition >20% and *p < *0.05: test condition *vs*. non-treated cells. All experiments were performed at least six times with two replicate experiments.

Further investigations were done on evernic acid (**1**) to better characterize and improve its anti-biofilm activity. The anti-maturation and anti-biofilm activities were determined at concentrations ranging between 1.5 and 50 µM. In these experiments, evernic acid was tested alone or included in a CD vectorized by a CNC in order to improve its solubility. **Figure 3** shows the results obtained against the maturation phase (**Figure 3A**) and against a mature biofilm (**Figure 3B**). CD/CNC was tested alone and loaded with evernic acid at an equivalent concentration.

However, IC_50_ was never obtained for anti-maturation tests using encapsulated evernic acid, regardless of incubation time.

For anti-biofilm tests, 50% inhibition of the biofilm was obtained after 48 h of contact with 12.5 µM evernic acid with or without cyclodextrin. The presence of cyclodextrin did not increase the evernic acid activity. Cyclodextrin alone never reached 50% inhibition of the mature biofilm.

## Discussion

The capacity of the studied compounds to limit biofilm growth and maturation just after the *C. albicans* adhesion phase was evaluated in order to identify molecules that could prevent *C. albicans* colonization of surfaces. The fact that the common dibenzofuran-like compound usnic acid (**11**) was not active at 200 µg/ml (**Table 1**) is in line with our previous results showing that lichen extracts containing a high percentage of usnic acid (*Usnea florida* and *Flavoparmelia caperata* extracts) were not active against the *C. albicans* maturation step (Millot et al., 2017; Dieu et al., 2020). Nevertheless, Nithyanand et al. reported that usnic acid was active against biofilm growth at 100 µg/ml, using another strain of *C. albicans* (ATCC: 90028) (65% of biofilm inhibition) and a different protocol (ring test). Contrary to our study, usnic acid was added to yeast before the adhesion step (Nithyanand et al., 2015b). We studied two types of depsidones. The first group corresponded to depsidones containing an additional lactone ring: stictic derivatives (**6**-**8**). In this group, the compounds were characterized by the absence of carboxyl functions and the presence of only one hydroxyl group. The second group included depsidones with additional aliphatic chains, represented by physodic and hydroxyphysodic acids (**9**–**10**). These compounds possess two to three phenolic groups as well as a carboxyl function. Two aliphatic chains also increase their lipophilicity. The stictic acid derivatives displayed no relevant anti-maturation activity (IC_50_ > 200 µg/ml). Despite their interesting additional chemical features, the same conclusion was made for physodic acid derivatives. The relationships between structure and the anti-biofilm activity of polyphenol compounds have been seldom explored up to now, and the rare, published hypotheses need to be confirmed—for example, Shahzad *et al*. compared the anti-maturation activity of several common polyphenols against *C. albicans* and found that curcumin was more active than pyrogallol. They attributed this superiority to the presence of two phenol groups connected by two α,β unsaturated carbonyl groups with unsaturated double bonds in the central connecting chain in curcumin instead of only one phenol group in pyrogallol (Shahzad et al., 2014). This hypothesis cannot be transposed to our study. Finally, the depside class is the most promising, with two compounds inducing a significant anti-maturation effect starting at 24 h of contact (squamatic acid **2** and thamnolic acid **3**; IC_50_ = 128 and 120 µM, respectively). Their effect increased after 48 h of contact: with IC_50_ reaching 32 and 30 µM, respectively. Interestingly, evernic acid **1** was the most active but required longer contact (IC_50_ 18 µM after 48 h of contact). Overall, activities after 48 h of contact were higher than those observed after 24 h. This observation is in line with the results of our previous study on lichen extracts (Millot et al., 2017). The comparison of anti-maturation activities of the five tested depsides suggested some hypotheses on the structure–activity relationships. Indeed for anti-maturation activity, the inactive atranorin (**4**) contains a methylated carboxyl function on ring B. On ring A, an aldehyde group and an additional hydroxyl group differ from compounds **1**–**2**. Thus, chemical features of atranorin are close to those of stictic derivatives of the depsidone group (**6**–**8**) (carboxyl group involved in cyclization in the lactone ring and presence of an aldehyde group) which were not active either.

The anti-biofilm activity of lichen compounds was also evaluated against a 24-h-old biofilm in order to identify new candidates for the destruction of the biofilm structure, providing the opportunity to increase the efficacy of conventional antifungal drugs. As for the anti-maturation results, dibenzofuran-like usnic acid and the depsidones displayed no anti-biofilm activity (**Table 1**). Contrary to what we observed, Peralta *et al*. showed that usnic acid displayed some anti-biofilm activity: in the presence of 4 μg/ml usnic acid, the biomass of a 48-h *C. albicans* biofilm was reduced to 4.91 μm^3^/μm^2^ compared to the control (18.39 μm^3^/μm^2^), corresponding to an inhibition of 73.3% (Peralta et al., 2016). The thickness was also reduced by 76.8%. However, the anti-biofilm methods (CSLM images) and *C. albicans* strains [resistant strain (12–99) and sensitive strain (2–76)] were different from those used in our study, which makes a comparison difficult. Usnic acid was also tested against other *Candida* species—for example, Kvasnickova *et al*. showed that usnic acid had no anti-biofilm properties (24-h biofilm) against *Candida krusei* and *Candida parapsilosis* even at a concentration of 300 μg/ml (Kvasnickova et al., 2015), whereas Pires et al. found that 31.2 and 62.5 µg/ml of this molecule induced 80% inhibition of cell growth compared to untreated controls of 24-h *Candida orthopsilosis* and *C. parapsilosis* biofilms, respectively (Pires et al., 2012). So, the differences in the methods make a comparison difficult, but usnic acid activity, overall, seems modest against *Candida* spp. biofilms.

Evernic acid with MBIC_50_ <12.5 µM was therefore the most active compound to target both the maturation phase and an already formed *C. albicans* biofilm, suggesting both curative and prophylactic interests. Its inhibitory activity was stronger after 48 h compared to 24 h, suggesting a delayed but persistent activity. This depside has two phenolic functions and at least one carboxyl group and differs from compounds **2** and **3** by the absence of a carboxyl function on ring A and an additional methyl group on ring B. It is also important to note that the ester linkage of depsides is brittle and can be easily hydrolyzed to give two monophenolic units. Nomura *et al*. reported the activity of depsides related to evernic acid and its monophenol derivatives against *L. pneumophila* biofilms (Nomura et al., 2013). Even if links have never been established between anti-biofilm inhibition of bacterial aggregates and fungal aggregates, Nomura et al. described a moderate anti-biofilm activity of evernic acid derivatives (23% inhibition at 4 µg/ml). The ability of evernic acid to modulate *P. aeruginosa* quorum sensing (Gökalsın and Sesal, 2016) has also been described, encouraging a thorough research investigation on depsides.

SEM observations of untreated biofilms confirmed the presence of yeast forms, hyphae, and matrix forming the *C. albicans* biofilms (**Figures 2, A1, A2**). Observation of biofilms treated with evernic acid highlighted some changes in cell morphology as well as the extracellular matrix. These changes were obvious at both 50 µg/ml (150 µM) and 100 µg/ml (300 µM) evernic acid but were more pronounced at the highest concentration. After treatment, some yeasts were wrinkled, and their matrix was more condensed with a less coating aspect. It seemed that the matrix radiated around the yeasts. Altogether our results suggest that evernic acid acted targeting especially the matrix. This hypothesis could at least partially explain its delayed and persistent activity and also its ability to act not only by counteracting the maturation of the biofilm but also by reducing an already formed biofilm.

The tested compounds displayed no significant activity against *C. albicans* growing plaktonically with MIC > 100 μg/ml (300 μM) [fluconoazole MIC: 4 μg/ml (13 μM)]. This result was not surprising as our previous studies never demonstrated that lichen extracts were active against planktonic *C. albicans* yeasts, whatever the species and solvent used (Millot et al., 2017). This was also in line with those of other teams who reported no antifungal activity for the tested depsides and depsidones through the disk diffusion method using 100 µg/ml (Yilmaz et al., 2004; Candan et al., 2006; Candan et al., 2007; Ranković et al., 2007; Studzińska-Sroka et al., 2015). For usnic acid (**11**), these results are in agreement with those obtained by Nithyanand *et al*. who also reported no antifungal activity against *C. albicans* even at 100 µg/ml (Nithyanand et al., 2015b).

Hemolysis and cell inhibition induced by the three depsides responsible for the highest anti-maturation or anti-biofilm effect were tested (**Table 2**). These depsides used at a concentration of 100 µg/ml induced weak red blood cell hemolysis ranging between 0 and 4%, thamnolic acid was associated with less hemolysis, and evernic acid had no effect at all. Overall, we can say that the seven tested compounds induced no more than 0.7% hemolysis. Regarding the inhibitory activity of the most promising depsides, squamatic and thamnolic acids (used at a concentration closer to that corresponding to the lowest anti-biofilm or anti-maturation MMIC_50_ 12.5 μg/ml or MBIC 6.25 μg/ml) did not prohibit HeLa cell growth by more than 22% whatever the depside. Furthermore, a high concentration (100 µg/ml) was responsible for the lowest inhibitory activities, slowing HeLa cell growth by 44%, whereas at the same concentration evernic acid checked growth by more than 70%. Despite the relatively high toxicity of evernic acid on HeLa cells, the antibiofilm activity is still valuable for lock therapy for medical devices which do not imply a contact with patients (Bouza et al., 2014; Imbert and Rammaert, 2018). Indeed synthesis or hemisynthesis modification could be realized in order to diminish the cytotoxicity.

In order to improve the solubility of evernic acid in a biological aqueous medium and to increase its anti-biofilm activity, this compound was included in a CD/CNC complex (Ndong Ntoutoume et al., 2016; Teodoro et al., 2017). The results (**Figure 3**) showed that encapsulation of evernic acid more or less maintained its activity against maturation phase or against already formed biofilms while the solubily in aqueous medium has been turned out.

In conclusion, a small group of lichen depsides and depsidones was tested against *C. albicans* biofilms. To our knowledge, this is the first report of anti-biofilm activity of such compounds against yeasts. Generally, depsides were more effective than depsidones in inhibiting the maturation phase during the biofilm formation. It can be argued that these molecules act as prodrugs through the formation of more active monophenol units against the biofilm. Further experiments on monomers must be considered. Among the tested depsides, evernic acid was the most promising for both prevention of biofilm formation and eradication of preformed biofilms. Indeed it was able to reduce the metabolic activity of *C. albicans* cells forming a 24-h-old biofilm with an IC_50_ of 37 µM. Modulation of evernic acid hydrosolubility could help increase its ability to penetrate the biofilm and thus its anti-biofilm activity, but its inclusion in cyclodextrin/cellulose nanocrystals gave inconclusive results. Other biological activities have been described for evernic acid, increasing the potential value of this compound—for example, recently, evernic acid was found to stabilize neuronal mitochondrial function and ameliorate neuro-inflammation-associated A1-astroglial activation (Lee et al., 2021). Further investigations are now needed, such as testing a panel of clinical strains isolated from various sites of patients with biofilm-related candidiasis or doing *in vivo* experiments. Our findings suggested that evernic acid could be a good candidate in the current search for new agents able to contribute to the prevention of biofilm growth and, in a more curative approach, to the eradication of already formed biofilms. These data could be useful in the development of new strategies for the treatment of biofilm-related candidiasis.

## Data Availability Statement

The datasets presented in this study can be found in online repositories. The names of the repository/repositories and accession number(s) can be found in the article/supplementary material.

## Author Contributions

MG, MM, LM, and CI contributed to the conception and design of the study. MM performed lichen compound extractions and identification, and GN and VS synthetized the complex with cylcodextrin, nanocrystal, and evernic acid. GH and J-LB performed the biofilm tests and SEM and statistical analyses. CJ performed the cytotoxicity assays. MM, LM, MG and CI wrote the first draft of the manuscript. All authors contributed to the article and approved the submitted version.

## Conflict of Interest

The authors declare that the research was conducted in the absence of any commercial or financial relationships that could be construed as a potential conflict of interest.

## Publisher’s Note

All claims expressed in this article are solely those of the authors and do not necessarily represent those of their affiliated organizations, or those of the publisher, the editors and the reviewers. Any product that may be evaluated in this article, or claim that may be made by its manufacturer, is not guaranteed or endorsed by the publisher.
